# A Support-Based Reconstruction for SENSE MRI

**DOI:** 10.3390/s130404029

**Published:** 2013-03-25

**Authors:** Yudong Zhang, Bradley S. Peterson, Zhengchao Dong

**Affiliations:** Brain Imaging Lab & MRI Unit, New York State Psychiatry Institute & Columbia University, New York, NY 10032, USA; E-Mails: petersob@nyspi.columbia.edu (B.S.P.); dongzh@nyspi.columbia.edu (Z.D.)

**Keywords:** parallel imaging, sensitivity encoding, magnetic resonance imaging, region of support, sensitivity maps, polynomial model, morphological operator

## Abstract

A novel, rapid algorithm to speed up and improve the reconstruction of sensitivity encoding (SENSE) MRI was proposed in this paper. The essence of the algorithm was that it iteratively solved the model of simple SENSE on a pixel-by-pixel basis in the region of support (ROS). The ROS was obtained from scout images of eight channels by morphological operations such as opening and filling. All the pixels in the FOV were paired and classified into four types, according to their spatial locations with respect to the ROS, and each with corresponding procedures of solving the inverse problem for image reconstruction. The sensitivity maps, used for the image reconstruction and covering only the ROS, were obtained by a polynomial regression model without extrapolation to keep the estimation errors small. The experiments demonstrate that the proposed method improves the reconstruction of SENSE in terms of speed and accuracy. The mean square errors (MSE) of our reconstruction is reduced by 16.05% for a 2D brain MR image and the mean MSE over the whole slices in a 3D brain MRI is reduced by 30.44% compared to those of the traditional methods. The computation time is only 25%, 45%, and 70% of the traditional method for images with numbers of pixels in the orders of 10^3^, 10^4^, and 10^5^–10^7^, respectively.

## 1. Introduction

Parallel imaging (PI) is one of the most important applications of the phased-array surface coils introduced to the field of MRI more than two decades ago [1]. PI makes use of the characteristic sensitivity distributions of the spatially separated receiver coils to provide an additional spatial encoding mechanism, in addition to the conventional phase encoding and frequency encoding required for MRI. This sensitivity-based encoding allows one to skip some phase encoding steps, speeding up the acquisition of the MRI data. Several PI methods have been proposed in the past decade, and they can be classified into two main types according to the working domains for the image reconstruction [2]. One is the *k*-space-based method, such as simultaneous acquisition of spatial harmonics (SMASH) and generalized autocalibrating partially parallel acquisitions (GRAPPA) [3], in which the missing phase encoding data points in *k*-space were synthesized by combining the sensitivity information of the coils and the acquired phase encoding and frequency encoding data [4]; The other is the image domain-based method, such as sensitivity encoding (SENSE) [5], in which the full image was recovered from the sensitivity maps, derived from scout images acquired during the patient setup, and the folded images reconstructed directly from the undersampled data of the individual coil components. In this paper, we focus our research on the latter, which is most widely used in clinical practice, and propose a rapid and accurate algorithm for the reconstruction of SENSE MRI.

Consider a simple 2D SENSE with an acceleration rate of *r*, in which every (*r*−1) phase encoding lines in the conventional fully sampled *k*-space were skipped [6]. According to the Nyquist theorem, the resultant images of the component coils would be folded in the dimension corresponding to the phase encoding direction. The basic principle of the SENSE technique is to unfold the images with the help of the sensitivity maps to recover the full images. The data acquired at *m* image points by an array of *c* coils can be expressed in the form of direct problem as a set of linear equations:

(1) $$S_{\rho }=b$$

where *S* is an *mc* × *n* sensitivity map, *ρ* is an *n* × 1 column vector denoting the desired full image, and *b* is an *mc* × 1 column vector whose elements are points in the folded images of the *c* coils. The reconstructed full image *ρ* can be obtained as an inverse problem by solving the above linear equations.

The traditional method for solving Equation (1) suffers from the following three shortcomings [7,8]: (i) The values of both *m* and *n* are of the order of 10^5^ , making the computation time of direct image reconstruction impractically long in clinical settings; (ii) It solved the equations for the entire FOV and did not take into account the region of support [9]; (iii) The estimation of the sensitivity map is vulnerable to noises, because it involves extrapolation from the brain area to the background area.

An iterative method was introduced recently for the fast reconstruction of the SENSE-based MRI [10]. This method significantly reduced computation time and memory storage. However, in this and previous techniques [11,12], the sensitivity maps were estimated for the full FOV and the algorithms of reconstruction were also based on the full FOV, without distinguishing regions in the brain and regions in the background. In fact, the background region often constitutes a significant portion of the FOV and is filled with noise. Therefore, the signals in the background should be taken as zeros as prior knowledge and further excluded from the image reconstruction for the benefits of both accuracy and speed. In this paper we proposed a method for accurate and rapid reconstruction of SENSE MRI. The method is based on the pixel-by-pixel algorithm for the simple SENSE model, and is applied on the ROS that is obtained by morphological operations such as opening and filling. Accordingly, the sensitivity map is estimated for the ROS without extrapolation to the whole FOV, thus avoiding considerable estimation errors. Finally, the method uses the ROS at the reconstruction stage to guide the equation solving procedures, and uses the accurate sensitivity maps to reconstruct the full image.

## 2. Method

### 2.1. The Simple SENSE Model

For the model of simple 2D SENSE with an acceleration factor *r*, the *k*-space data were regularly undersampled by skipping every *r*—1 phase encoding lines in the full *k*-space [13]. Without losing generality, we consider as an example the case of acceleration factor of *r* = 2 and the number of coils *c* = 8. For a pixel at location (*x*, *y*) in the aliased image, the signals measured by each of the *eight* coils are given by following equations:

(2) $$\begin{array}{l} S_{1}(x,y)\rho (x,y)+S_{1}(x+\frac{\text{FOV}}{2},y)\rho (x+\frac{\text{FOV}}{2},y)=b_{1}(x,y)\\ \cdots \\ S_{8}(x,y)\rho (x,y)+S_{8}(x+\frac{\text{FOV}}{2},y)\rho (x+\frac{\text{FOV}}{2},y)=b_{8}(x,y) \end{array}$$

**Figure 1.**
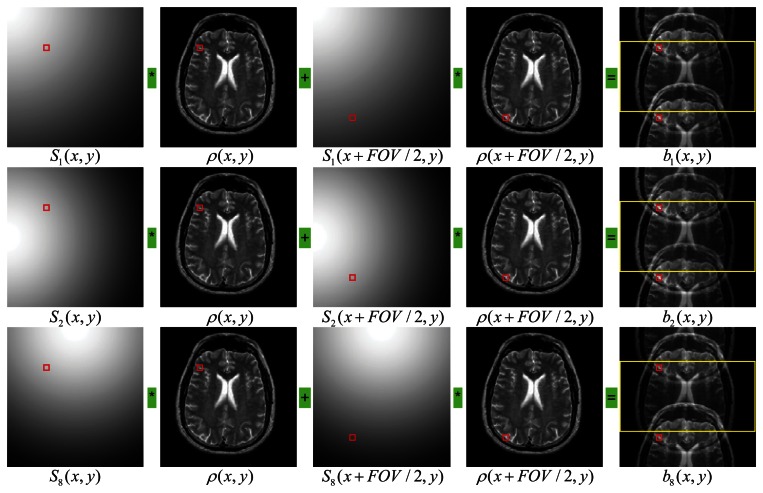
A pictorial illustration of the SENSE principles. The k-space is scanned every other row. The rows are for the first, the second and the eighth channels, respectively. Column 1 & 3 are the sensitivity maps, column 2 & 4 are the brain or the images to be reconstructed, and final column is the aliased brain images acquired by the corresponding channels. The red square marker denotes the pixel at location (*x*, *y*).

This is a simplified version of the direct problem previously given in Equation (1), which states that the signals at the locations of the two pixels (*x*, *y*) and (*x*+FOV/2, *y*) are aliased to generate the measured signals in the component coils. The illustration of SENSE is shown pictorially in Figure 1.

### 2.2. Iterative Method

There are three approaches to SENSE reconstruction [5]: (i) solving Equation (1) directly, which would take enormous storage and time; (ii) line-by-line iterations, in which each iteration estimates two rows of the original image; (iii) pixel-by-pixel iterations, recovering two pixels of the original image in each iteration.

**Figure 2.**
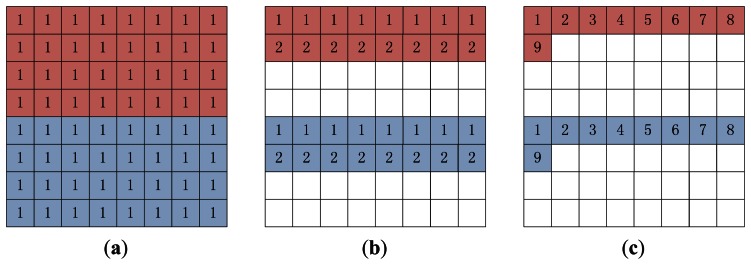
Schematic illustration of the three approaches to the SENSE reconstruction. The acceleration rate is 2. The upper 4 × 8 (pink) and the lower 4 × 8 (blue) matrices in the above represent the voxels in the aliased images. The full image is an 8 × 8 matrix. (**a**) Non-iterative method: we unfold the aliased images to obtain the full image in one iteration; (**b**) Line-by-line iteration method: we obtain the estimated voxels of the 1^st^ and the 5^th^ rows in the first iteration, and the 2^nd^ and the 6^th^ rows in the 2^nd^ iteration, and so on; (**c**) Pixel-by-pixel method: we obtain the estimated voxels in pairs. The digits *i* in the image denotes the voxel is estimated at the *i*-th iteration.

We briefly analyze the three approaches to recovering an 8 × 8 matrix from two aliased 4 × 8 images acquired with an acceleration factor of 2 (Figure 2). The first approach is non-iterative, and we obtain the estimate of the whole image without iteration. With the line-by-line iteration, we unfold the first lines in the aliased images to obtain the first and fifth row of the full image at the 1^st^ iteration, and then we unfold the second lines in the aliased images to obtain the second and sixth row at the 2^nd^ iteration, and so on. With the pixel-by-pixel iteration, we obtain the *ρ*(1, 1) and *ρ*(5, 1) at the 1^st^ iteration, the *ρ*(1, 2) and *ρ*(5, 2) at the 2^nd^ iteration, until the *ρ*(1, 8) and *ρ*(5, 8) at the 8^th^ iteration; Then the scan turns to the next row, and we obtain the *ρ*(2, 1) and *ρ*(6, 1) at the 9^th^ iteration, and so on. In this paper, we chose the pixel-by-pixel iteration since it only requires least memory.

In matrix notation, equations in Equation (2) can be expressed in Equation (3), where *S* is an 8 × 2 matrix whose elements are sensitivities of the coil components at two locations of (*x*, *y*) and (*x* + FOV/2, *y*), and *b* is a column vector of 8 elements, representing the superimposed signals from the two voxels at (*x*, *y*) and (*x*+FOV/2, *y*) recorded by the eight coil components. The two intensities of *ρ*(x, y) and *ρ*(*x*+FOV/2, *y*) were obtained by solving the following equations:

(3) $$\begin{array}{l} S_{8\times 2}\left[\begin{array}{c} \rho (x,y)\\ \rho (x+\frac{\text{FOV}}{2},y) \end{array}\right]=b_{8\times 1}\\ S_{8\times 2}=\left[\begin{array}{cc} S_{8\times 2}^{1} & S_{8\times 2}^{2} \end{array}\right] \end{array}$$

Here *S*^1^ and *S*^2^ are two column vectors of eight elements. In essence, the solution of Equation (3) is a procedure of recovering the two voxels at (*x*, *y*) and (*x*+FOV/2, *y*), by unfolding the superimposed signals. We will show that the entire image can be reconstructed when all the superimposed signals within the region of support (ROS) are unfolded. In the following subsections we introduce the ROS, give the detailed procedures for estimating sensitivity map *S* based on ROS, and describe the ROS-based SENSE reconstruction.

### 2.3. Region of Support

**Figure 3.**
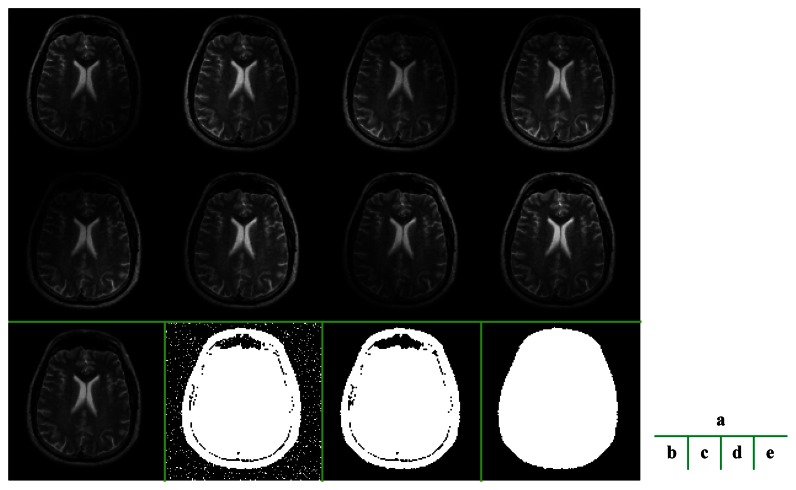
Illustration of obtaining ROS: (**a**) eight Scout images; (**b**) the summation of the squares of the magnitudes of eight scout images; (**c**) Support area, use 1% of maximum intensity as the threshold; followed by two morphological operations: (**d**) ROS after opening Procedures; (**e**) ROS after filling Procedures.

The ROS is detected from the scout images of eight channels [14] (Figure 3). Let *O_i_* denotes the scout image of the *i*th channel, the ROS is calculated from following procedures.

(1) Calculate the power image defined as:
  (4) $$E=\sum_{i=1}^{8}O_{i}^{2}$$
(2) Find the support area. Here we choose the threshold as the 1% of maximum intensity value:
  (5) $$T_{1}=E>0.01\times \max(E)$$
(3) Perform the opening operation to eliminate the noise artifacts:
  (6) $$T_{2}=\mathrm{open}(T_{1})$$
(4) Use the filling method of mathematical morphologic operations to fill the holes:
  (7) $$\text{ROS}=\text{fill}(T_{2})$$

The mean essence of the algorithm is thresholding based on the signal-to-noise ratio (SNR), because the brain area usually contains higher signal than the background does. But other morphological operations are also necessary to ensure an example of these procedures is illustrated in Figure 3. The final extracted ROS was very close to the shape of the brain.

### 2.4. Estimation of Sensitivity Maps

In the literature [9,10], sensitivity maps were generated for the whole FOV by fitting and extrapolating the raw sensitivity maps obtained from low resolution scout images acquired in a fast reference scan. Sensitivity maps in the whole FOV facilitate the whole FOV-based reconstruction of SENSE MRI, but they are difficult to estimate accurately not only in the background region but also within the brain, because the extrapolation inevitably introduces errors. We therefore adopted an ROS-based approach to accurately estimating sensitivity maps.

We used a second-order polynomial regression model to approximate the realistic sensitivity map and to reduce noises. The model is as follows:

(8) $$S(x,y)=a_{20}x^{2}+a_{11}xy+a_{02}y^{2}+a_{10}x+a_{01}y+a_{00}$$

Let *P* = [*x*, *y*] denote the point at position [*x*, *y*], model(*P*) = [*x*^2^, *xy*, *y*^2^, *x*, *y*, 1] denote the polynomial model of point *P*, and *A* = [*a*_20_, *a*_11_, *a*_02_, *a*_10_, *a*_01_, *a*_00_]^T^ denote the predictor vector (regressor), and then Equation (8) can be rewritten as:

(9) S(P)=model(P)×A

Suppose there are *r* pixels in ROS, the estimated sensitivity map can be obtained by solving the following equations:

(10) $${\left[\text{model}(\text{ROS})\right]}_{r\times 6}A{\left(i\right)}_{6\times 1}={\left[D{\left(i\right)}_{\text{ROS}}\right]}_{r\times 1}$$

Here model(ROS)={;model(P)|P ∈ ROS}. *D*(*i*) denotes the energy ratio of the of *i*-th scout image to the whole of scout images:

(11) $$D(i)=\frac{O^{2}(i)}{\sum_{i=1}^{8}O^{2}(i)}$$

Finally, the regressor *A*(*i*) was obtained by solving Equation (10). The estimated sensitivity map covering the full image *S*(full) was inferred from regressor *A* and design matrix model(full):

(12) S(full)=model(full)×A

Here S(full)= {S(P)|P ∈ full}, and model(full)= {model(P)|P ∈ full}. However, the regressor *A* was obtained only from samples of ROS as seen in Equation (10), so the *S*(full) implied that there should be extrapolation out of ROS, which would cause errors. However, the estimation of the sensitivity map covering only the ROS (Equation (13)) involved only interpolation and may be more accurate:

(13) S(ROS)=model(ROS)×A

**Figure 4.**
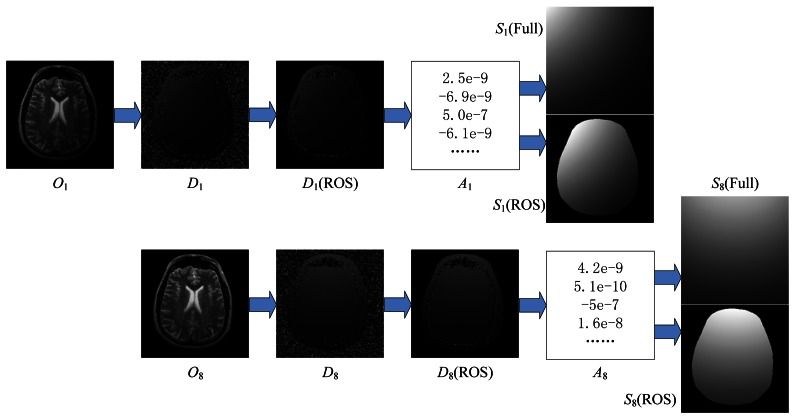
Estimation of sensitivity maps. From left to right columns: eight scout images (O_1_ to O_8_) from eight channels; energy ratios (D_1_ to D_8_); Ds within ROS; regressor parameters by Equation (10); the estimated sensitivity maps *S_i_* (shown also the *S_i_* over the full FOV).

Here S(ROS)= {S(P)|P ∈ROS}. An example of the estimation of sensitivity maps is illustrated in Figure 4.

### 2.5. ROS-Based Reconstruction

For the ROS-based rapid algorithm of SENSE reconstruction, we classified the pair of aliasing pixels into four groups as follows:

(14) $$\left[\begin{array}{cc} \rho (x,y) & \rho (x+\frac{\text{FOV}}{2},y) \end{array}\right]=\{\begin{array}{ll} \left[\begin{array}{cc} \rho (x,y) & \rho (x+\frac{\text{FOV}}{2},y) \end{array}\right] & (x,y)\&(x+\frac{\text{FOV}}{2},y)\in \text{ROS}\\ \left[\begin{array}{cc} \rho (x,y) & 0 \end{array}\right] & (x,y)\in \text{ROS}\\ \left[\begin{array}{cc} 0 & \rho (x+\frac{\text{FOV}}{2},y) \end{array}\right] & (x+\frac{\text{FOV}}{2},y)\in \text{ROS}\\ \left[\begin{array}{cc} 0 & 0 \end{array}\right] & \text{otherwise} \end{array}$$

(1) Both pixels fall into the ROS. We solve Equation (3) to get *ρ*(*x*, *y*) and *ρ*(*x*+FOV/2, *y*).
(2) The point of (*x*+FOV/2, *y*) falls into the ROS. We assign 0 to *ρ*(x, y), and the Equation (3) can be transformed to the following:
  (15) $$S_{8\times 1}^{2}\rho (x+\frac{\text{FOV}}{2},y)=b_{8\times 1}$$
  Afterwards, we calculate the intensity value of *ρ*(x + FOV/2, *y*) by solving Equation (15). The linear coefficients matrix *S*^1^ of Equation (15) is 8 × 1, only half of the size of the linear coefficients matrix *S* of Equation (3).
(3) The point of (*x*, *y*) falls into the ROS. Similarly, we assign 0 to *ρ*(*x* + FOV/2, *y*) and transform Equation (3) to the following,
  (16) $$S_{8\times 1}^{1}\rho (x,y)=b_{8\times 1}$$
  Afterwards, we solve Equation (16) to get the intensity value of pixel *ρ*(*x*, *y*).
(4) Neither of the pair falls into the ROS. We assign a value of 0 to each of the pixels. As seen from Equations (14)–(16), recovering the pixels of groups 1 to 3 is straightforward and only requires simple arithmetic operations. Only the recovery of pixels of group 4 needs matrix operation as in the traditional methods. Therefore, our proposed method is computationally simple and can be executed rapidly.

## 3. Experiments

We carried out experiments to assess the performance of our method and, in particular, to compare our method with the traditional methods. The experiments were carried out on the platform of Windows XP on a desktop PC rquipped with an Intel Pentium 4, 3 GHz processor and 2 GB memory. The programs were developed via Matlab 2010b.

### 3.1. Comparison of the Algorithms

**Figure 5.**
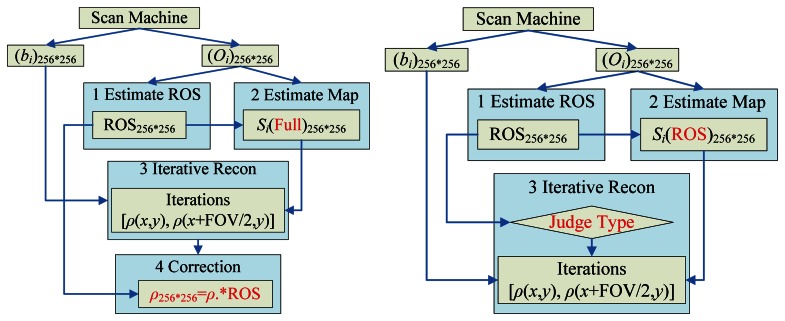
Flowchart of (**a**) ROS-based correction; (**b**) ROS-based reconstruction (the differences are labeled with red color), please see the paragraph below for the details.

The traditional method used ROS as a correction tool, viz., to multiply final reconstruction results with the ROS to reduce the background noises. We call it “ROS-based correction” for short. In our method, we used the ROS at the reconstruction stage to group the pairs into different types, which is referred to as “ROS-based reconstruction”. The main differences of ROS-based correction and ROS-based reconstruction lie in the following three points (shown in the red font in Figure 5):

(1) ROS-based correction calculates the sensitivity map of the full image, which involves extrapolation; however, ROS-based reconstruction only needs the sensitivity map within the ROS, which only involves interpolation.
(2) ROS-based correction directly solves Equation (3) no matter the pixel pairs locate in the ROS or in the background. Conversely, the ROS-based reconstruction classifies Equation (3) into four types, and solves different type by different methods, which greatly hasten the procedures.
(3) ROS-based correction needs to correct the final result by multiplying it with the ROS, while the ROS-based reconstruction is free from this procedure.

### 3.2. The Quality of the Reconstruction

We compared our proposed ROS-based reconstruction method with traditional ROS-based correction method. We first used a fully sampled T2 weighted brain MRI image as a reference data set, undersampled the data with an acceleration rate of 2, applied the aforementioned two methods to reconstruct the images from the undersampled data, and compared the results of the two methods using mean absolute error (MAE) and mean square errors (MSE), which were calculated against the ground truth. To facilitate fair comparison, we calculated MAEs and MSEs on the ROS instead of the whole FOV. In order to assess the performance of sensitivity map estimation, we added Gaussian noise of zero mean and 10^−4^ variance, which indicates the pixels in the image will have a fluctuation of $256\times \sqrt{10^{-4}}=\pm 2.56$ in their gray intensity values.

**Figure 6.**
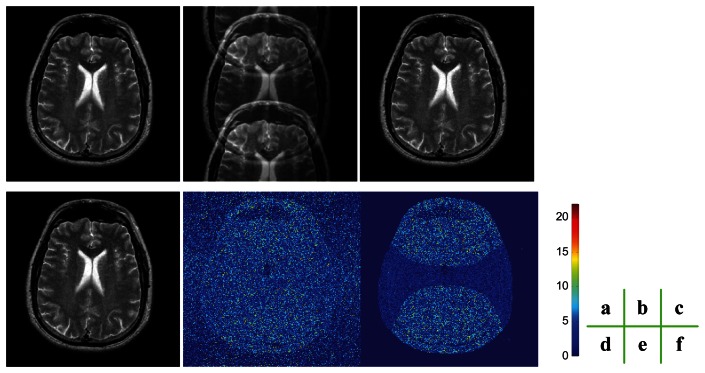
Comparison of the quality of the reconstructions: (**a**) Original, fully sampled T_2_-weighted brain MR image; (**b**) The aliased image; (**c**) Reconstructed image by ROS-based correction; (**d**) Reconstructed image by ROS-based reconstruction; (**e**) Difference between (c) and (a); (**f**) Difference between (d) and (a).

Figure 6 indicates that the noise influenced the estimation of sensitivity maps as the artifacts near the center of the brain. The detailed errors indicate that the MAE and MSE of our method are 1.6988 and 9.4573, respectively, while the MAE and MSE of ROS-based correction method 2.0162 and 11.2656, respectively. The results render a reduction of 15.07% for MAE and a reduction of 16.05% for MSE by the proposed method. The errors are calculated on the FOV area, not including the background.

The reason why our ROS-based reconstruction method can achieve less error leans on the H-shape area in Figure 6(f). We know *a priori* that the corresponding pixels of “H” area should be zero, and the pixels are recovered in pairs. Therefore, we can get a higher signal-to-noise ratio on the H-shape area due to the *a priori* information.

**Figure 7.**
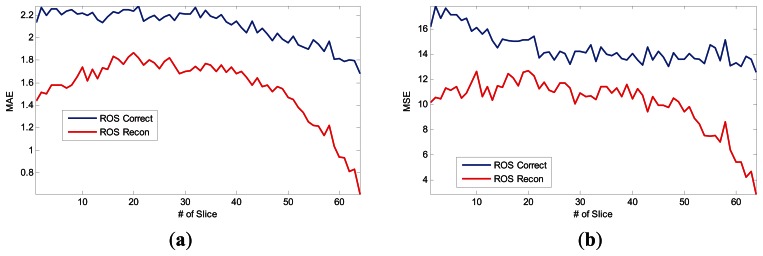
3D Brain Reconstruction Results: (**a**) MAE curve, (**b**) MSE curve. X-axis denotes the index of brain slices, and y-axis denotes the error values.

The advantages of our ROS-based reconstruction method over the conventional method are more significant for 3D MRI than for 2D MRI. We used a 128 × 128 × 64 MRI data and added Gaussian noise with zero mean and 10^−4^ variance. The curves of MAE and MSE of two methods *versus* different number of slices are depicted in Figure 7. The mean MAE and MSE of the whole slices for the ROS-based correction method are 2.1062 and 14.5392 and, conversely, the mean MAE and MSE of ROS-based reconstruction method are only 1.5519 and 10.1134. The results represent 26.32% reduction of mean MAE and 30.44% reduction of mean MSE.

### 3.3. Computation Time

**Figure 8.**
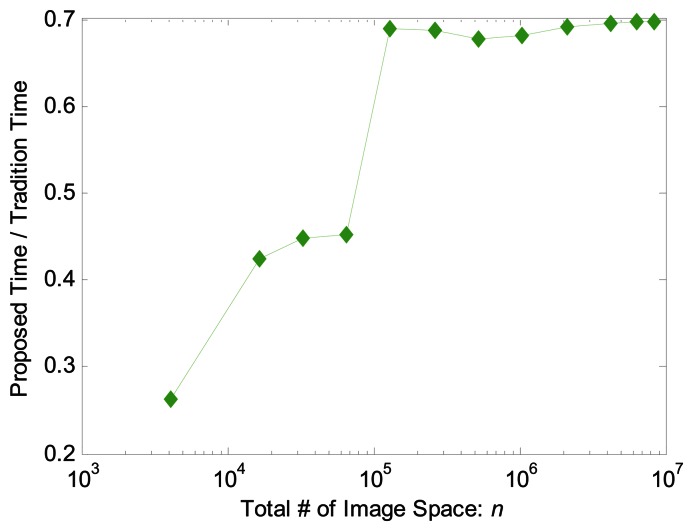
Comparison of the computation times between the proposed method and the traditional method. The x-axis denotes the total number of image voxels, and the y-axis denotes the ratio of computation times of the proposed method to traditional method. We used logarithm scale for clearance.

We compared the computation times of our method and the conventional method using datasets of 2D/3D MRI, 3D MRSI, 3D DTI and 3D fMRI. The 1^st^ 3 dimensions are spatial dimensions and the 4^th^ for 3D MRSI, 3D DTI and fMRI are spectral, angular and temporal respectively. For images with small size, e.g., a 2D MRI of 256 × 256, the computation time of the proposed method is less than half of the conventional method (Table 1). When the size of the images increases, the ratio of computation times of the current method to conventional method increases, but the gain of the acceleration is significant. In the case of 3D fMRI, for example, the current method shortened the reconstruction time from more 12 min to less than 8.5 min. We further increased the size of the images and found the aforementioned ratios remained <0.7 (Figure 8).

## 4. Conclusions

In this study, we have proposed an ROS-based method for the reconstruction of SENSE MRI. The method involves an ROS-based accurate estimation of sensitivity maps and an ROS-based pixel-by-pixel iterative algorithm for the reconstruction. The experiments show that the method is fast and significantly improve the quality of reconstruction of SENSE MRI.

The MSE of our reconstruction is reduced by 16.05% for a 2D brain MR image and the mean MSE over the whole slices in a 3D brain MRI is reduced by 30.44% compared to those of the traditional methods. The computation time is only 25%, 45%, and 70% of the traditional method for images with numbers of pixels in the orders of 10^3^, 10^4^, and 10^5^–10^7^, respectively.

However, the computation advantage of our method depends on the support size of the brain in the FOV. If the brain occupies most of the FOV, the computational advantage will be compromised. Therefore, our method is suitable for images which contain large background area.

One of an interesting future work will be on the combination of ROS and other techniques, such as the 3D wavelet representation [15] that handles a 3D dataset as a whole and address reconstruction artifacts efficiently.

## Figures and Tables

**Table 1.** Comparison of computation times image reconstruction by the conventional and the proposed methods for different MR modalities (*c* = 8, *r* = 2).

**Type of Image**	**Size of images**	**No. of image pixels n**	**Time (second)**	

			**Traditional method**	**Proposed method**
2D MRI	256 × 256	65,536	5.7051	2.5774
3D MRI	128 × 128 × 64	1,048,576	91.4645	62.2536
3D MRSI	32 × 32 × 8 × 512	4,194,304	365.9453	254.6059
3D DTI	128 × 128 × 16 × 24	6,291,456	549.7143	383.4347
3D fMRI	64 × 64 × 32 × 64	8,388,608	735.9560	512.3298
